# Robotic colorectal surgery in elderly patients: A single‐centre experience

**DOI:** 10.1002/rcs.2431

**Published:** 2022-06-26

**Authors:** Enda Hannan, Gerard Feeney, Mohammad Fahad Ullah, Eoghan Condon, John Calvin Coffey, Colin Peirce

**Affiliations:** ^1^ Department of Colorectal Surgery University Hospital Limerick Dooradoyle Limerick Ireland; ^2^ School of Medicine University of Limerick Dooradoyle Limerick Ireland

**Keywords:** elderly patients, geriatric surgery, older patients, robotic colorectal surgery

## Abstract

**Introduction:**

The purpose of this study was to evaluate outcomes in elderly patients (age ≥ 65 years) undergoing robotic colorectal surgery (RCRS) in comparison with non‐elderly patients.

**Materials and Methods:**

Data was collected on elderly and non‐elderly patients who underwent RCRS from a prospectively maintained database.

**Results:**

A total of 89 elderly and 73 non‐elderly patients were identified. No statistically significant differences in postoperative complication, reoperation, wound infection, anastomotic leak or mortality were observed. The median length of stay was 1 day longer in elderly patients (*p* = 0.007). Subgroup analysis of octogenarians demonstrated outcomes that compared favourably with younger patients.

**Conclusion:**

RCRS in elderly patients is safe and effective, with outcomes that do not differ significantly with younger patients. Older age should not be considered to be a specific exclusion criteria for RCRS. To our knowledge, this study represents the largest in the literature to examine outcomes specifically in elderly patients undergoing RCRS.

## 1 INTRODUCTION

The elderly population is expanding exponentially.^1^ By 2030, those aged 65 years or older will account for approximately 20% of the population in the United States.^1^ By this time, it is also estimated that the population over the age of 75 years will triple and the population over the age of 85 years will double.^2^ Colorectal cancer (CRC) has an incidence that increases with age, with a peak between the 7^th^ and 8^th^ decades.^3^, ^4^ Diverticular disease is also more common in older age, with a prevalence as high as 65% by the age of 85 years.^5^ Similarly, it has been shown that inflammatory bowel disease (IBD) is increasingly common in elderly patients.^6^ For these reasons, as life expectancy has increased, a growing number of elderly patients will present with colorectal disease that may require complex intra‐abdominal surgery.^7^ This is concerning for colorectal surgeons, as elderly patients provide significant perioperative and postoperative challenges due to an increased incidence of cardiovascular and pulmonary comorbidities which render them vulnerable to surgical morbidity and mortality.^8^

The open approach to colorectal surgery has been shown to be associated with significant morbidity and a slow postoperative recovery, with age being an independent risk factor for postoperative complication.^9^ Laparoscopic colorectal surgery (LCRS) offers numerous advantages to open surgery, such as a reduction in postoperative pain, inpatient length of stay and wound complications, whilst also offering an earlier return to normal activity.^10^ It has been shown by systematic review that LCRS offers a statistically significant reduction in mortality for elderly patients compared with an open approach.^4^, ^11^ However, LCRS is not without limitations and include an unstable assistant‐dependent view, exaggerated tremor, limited ergonomics and reduced dexterity.^12^ These limitations become particularly apparent in rectal surgery, where crowded instruments may clash in the confines of the narrow bony pelvis.^12^ For these reasons, reasonably high rates of conversion from LCRS to open surgery have been observed, and thus the benefits offered by a minimally invasive approach are lost.^13^

Robotic colorectal surgery (RCRS) is now favoured by many surgeons due to the inherent characteristics of robotic surgical platforms which allow surgeons to overcome the limitations of laparoscopic surgery.^14^ These include a stable surgeon‐controlled view, tremor elimination, improved ergonomics and greater instrument range of motion.^14^ These features are particularly attractive in rectal surgery, allowing for improved vision and dexterity in the highly challenging operative field of the pelvis.^14^ It has been shown that RCRS has comparable safety and efficacy to LCRS.^15^, ^16^ Such ergonomic advantages may allow surgeons to more consistently deliver the inherent benefits of minimally invasive surgery to elderly patients by avoiding the morbidity associated with conversion to open surgery due to technical difficulty.^17^, ^18^ However, there have been some reservations regarding the utilisation of RCRS in this patient population due to a hypothetical higher risk posed by a longer operative time with the maintenance of Trendelenburg position and prolonged pneumoperitoneum.^19^ Despite such concerns, there is currently limited evidence available on outcomes relating specifically to older populations undergoing RCRS.^20^, ^21^, ^22^ The purpose of this study was to investigate the safety, feasibility and efficacy of RCRS in elderly patients by comparing perioperative and postoperative outcomes in this cohort to a younger patient population in our institution, a tertiary referral university teaching hospital with a RCRS programme established in 2016.

## 2 MATERIALS AND METHODS

### 2.1 Patient selection & data collection

A retrospective analysis of a prospectively maintained database comprising all patients who underwent RCRS in our institution was performed. Demographic, perioperative, postoperative and surgical specimen data was collected. All patients who underwent RCRS for both benign and malignant pathology were included. While the definition of what constitutes an ‘elderly’ patient is controversial, this has conventionally been considered age 65 and older, and this is the age at which a patient may be considered for referral to geriatric medicine in our healthcare service.^23^ Thus, patients aged 65 years and older were categorised as elderly, and those under the age as 65 years were categorised as non‐elderly. All patients diagnosed with CRC were discussed at the colorectal multidisciplinary team meeting prior to surgical intervention.

### 2.2 Surgical techniques

All operations were performed by fellowship‐trained consultant colorectal surgeons on the specialist division of the medical register who had completed proctorship programmes in RCRS using the da Vinci^®^ Xi dual console robotic surgical system (Intuitive Surgical Inc). Port placement was based on principles outlined in the manufacturer guidelines with four trocars placed in a line perpendicular to the target anatomy at a range 6–10 cm apart, with a further assistant port placed an appropriate distance away from the da Vinci^®^ ports. In most cases, a medial‐to‐lateral approach to dissection was performed with ligation of the relevant vasculature. A lateral‐to‐medial approach was very occasionally utilised if this initial approach was deemed unsafe or difficult.

For rectal resections, the specimen was extracted via a suprapubic incision and transanal stapling was performed to create the anastomosis in most cases, either by intracorporeal or extracorporeal technique based on surgeon preference and what was deemed safest for the patient intraoperatively. Other left‐sided resections, including left hemicolectomies and sigmoid colectomies were performed based on similar principles. For right hemicolectomies, anastomosis was performed in an extracorporeal fashion, varying between stapled and handsewn techniques according to individual surgeon preference.

### 2.3 Statistical analysis

Statistical analysis was performed using IBM SPSS version 24 (SPSS Inc). Continuous variables were reported by median value and interquartile range (IQR). Categorical variables were reported by their frequency and percentage calculated. Univariate analysis was performed using a Student's *t* test or Mann Whitney *U* test for continuous variables, and a Fischer's exact test for categorical variables. A *p*‐value of less than 0.05 was considered statistically significant without adjustment for the number of outcomes tested. The 95% confidence interval (95% CI) between exposure comparisons was calculated using the Bonnet‐Price difference of medians for continuous variables and the Wilson method for categorical variables. All procedures followed were in accordance with the ethical standards of the responsible committee on human experimentation (institutional and national) and with the Helsinki Declaration of 1975, as revised in 2000. As this was a retrospective service evaluation involving anonymised data, ethics committee approval was not required in our institution.

## 3 RESULTS

### 3.1 Baseline characteristics (Table 1)

**TABLE 1:** Patient demographics

	Overall (*n* = 162) *N* (%)	Elderly (*n* = 89) *N* (%)	Non‐elderly (*n* = 73) *N* (%)	*p*‐value	95% confidence interval [post‐estimate of difference]
Median age (years, [IQR])	63 (IQR 16) Min‐max: 25–89	72 (IQR 11.5) Min‐max: 65–89	56 (IQR 14) Min‐max: 25–64	<0.001	18, 23 (%) [16]
Gender					
Male	89 (54.9%)	51 (57.3%)	38 (42.7%)	0.25	−21, 10 (%) [13]
ASA grade					
I	23 (14.3%)	7 (7.9%)	16 (21.9%)	0.002	3, 25 (%) [9]
II	102 (62.9%)	56 (62.9%)	49 (67.2%)		−11, 19 (%) [7]
III	37 (22.8%)	26 (29.2%)	8 (10.9%)		−31, −6 (%) [18]
Body mass index					
Obese	42 (25.9%)	27 (30.3%)	15 (20.5%)	0.07	−23, 04 (%) [12]
Malignant versus benign pathology					
Malignant	104 (64.2%)	64 (71.9%)	40 (54.8%)	0.01	−32, −2 (%) [24]
Previous abdominal surgery					
Midline laparotomy	17 (10.5%)	10 (11.2%)	7 (9.6%)	0.7	−11, 8 (%) [3]
Surgical procedure					
Right hemicolectomy	52 (32.1%)	31 (34.8%)	21 (28.8%)	0.07	−2, 8 (%) [10]
Extended right hemicolectomy	4 (2.5%)	2 (2.2%)	2 (2.7%)		−4, 5 (%) [0]
Ileocolic resection	4 (2.4%)	0 (0%)	4 (5.5%)		1, 10 (%) [4]
Caecectomy	1 (0.7%)	0 (0%)	1 (1.4%)		−1, 4 (%) [1]
Left hemicolectomy	6 (3.7%)	6 (6.7%)	0 (0%)		−13, −1 (%) [0]
Sigmoid colectomy	9 (5.5%)	5 (5.6%)	4 (5.5%)		−7, 7 (%) [1]
Anterior resection	59 (36.4%)	30 (33.8)	29 (39.7%)		−9, 21 (%) [1]
Abdominoperineal resection	13 (8%)	8 (9%)	5 (6.8%)		−11, 6 (%) [3]
Hartmann’s procedure	7 (4.3%)	6 (6.8%)	1 (1.4%)		−11, 1 (%) [5]
Completion proctectomy	6 (3.7%)	1 (1.1%)	5 (6.8%)		−1, 12 (%) [4]
Reversal of Hartmann’s procedure	1 (0.7%)	0 (0%)	1 (1.4%)		−1, 4 (%) [1]
Pathological T stage (n = 104)					
0	6 (5.8%)	0 (0%)	6 (15%)	<0.001	2, 14 (%) [6]
1	7 (6.7%)	1 (1.6%)	6 (15%)		1, 13 (%) [5]
2	23 (22.1%)	18 (28.1%)	5 (12.5)		−24, −3 (%) [13]
3	51 (49%)	32 (50%)	19 (47.5%)		−24, 4 (%) [13]
4	17 (16.3%)	13 (20.3%)	4 (10%)		−18, 1 (%) [9]

Abbreviations: IQR, interquartile range; Min‐max, minimum value to maximum value.

Between July 2016 and July 2021, 162 patients underwent RCRS in our institution. The majority (54.9%, *n* = 89) were in the elderly cohort. The median age of this cohort was 72 years, of which 57.3% (*n* = 51) were male. The American Society of Anaesthesiologists (ASA) grade was 3 in 29.2% (*n* = 26) of patients in this group, while 30.3% (*n* = 27) had a body mass index (BMI) that classified them as obese (≥30.0 kg/m^2^). The majority of operations performed in elderly patients were either anterior resections (33.8%, *n* = 30) or right hemicolectomies (34.8%, *n* = 31) and most patients (71.9%, *n* = 64) underwent surgery for CRC.

During the same period, 73 patients who underwent RCRS were less than 65 years of age. Of these, 42.7% (*n* = 38) were male and the median age was 56 years. The ASA grade was 3 in 10.9% (*n* = 8) of patients in this cohort, while 20.5% (*n* = 15) were obese. Most underwent either anterior resection (39.7%, *n* = 29) or right hemicolectomy (28.8%, *n* = 21) and the most common indication for surgery was CRC (54.8%, *n* = 40).

### 3.2 Perioperative outcomes (Table 2)

**TABLE 2:** Perioperative & postoperative outcomes

Perioperative outcomes				
	Elderly (*n* = 89) *N* (%)	Non‐elderly (*n* = 73) *N* (%)	*p*‐value	95% confidence interval [post‐estimate of difference]
Median operative time (minutes)	228 (IQR 104)	254 (IQR 105)	0.09	−9, 46 (min) [26]
Median docking time (minutes)	30 (IQR 15)	34 (IQR 17)	0.06	−11, 3 (min) [4]
Median estimated blood loss (ml)	100 (IQR 95)	80 (IQR 152.5)	0.4	−68, 40 (ml) [20]
Conversion to open surgery (%)	3 (3.4%)	3 (4.1%)	0.4	−5, 7 (%) [0]

Postoperative outcomes				
	Elderly (*n* = 89) *N* (%)	Non‐elderly (*n* = 73) *N* (%)	*p*‐value	95% confidence interval
Median LOS (days)	7 (IQR 9) Min‐max: 4–81	6 (IQR 10) Min‐max: 3–29	0.007	−7, −1 (days) [1]
Postoperative complication (%)	27 (30.3%)	19 (26%)	0.2	−18, 9 (%) [8]
Major morbidity (%)	6 (6.7%)	9 (12.3%)	0.1	−3, 15 (%) [3]
Cardiorespiratory complication (%)	1 (1.1%)	0 (0%)	0.2	−4, 1 (%) [1]
SSI (%)	15 (16.9%)	7 (9.6%)	0.09	−18, 3 (%) [8]
Anastomotic leak (%)	2 (2.2%)	5 (6.8%)	0.07	−2, 11 (%) [3]
30‐day reoperation (%)	5 (5.6%)	7 (9.6%)	0.2	−4, 12 (%) [2]
30‐day readmission (%)	3 (3.4%)	2 (2.7%)	0.4	−6, 5 (%) [1]
30‐day mortality (%)	1 (1.1%)	0 (0%)	0.2	−4, 1 (%) [1]
Median lymph node yield	14	14	1.0	−8, 15 (%) [0]
R1 margins (%)	0 (0%)	1 (1.4%)	0.1	−1, 4 (%) [1]

Abbreviations: IQR, interquartile range; Min‐max, minimum value to maximum value.

The median operative time in the elderly cohort was 228 min (IQR 104 min), with a median time from commencing surgery to docking the robot of 30 min (IQR 15 min). In the non‐elderly cohort, the median operative time was 254 min (IQR 105 min) with a median docking time of 34 min (IQR 17 min). The median estimated blood loss was 100 ml (IQR 105 ml) in elderly patients and 80 ml (IQR 152.5 ml) in non‐elderly patients (*p* = 0.4, 95% CI −68 to 40). Conversion to open surgery was defined as an unplanned midline laparotomy due to inability to complete a planned robotic stage of the operation. The incidence of conversion from robotic surgery to open surgery was 3.4% (*n* = 3) in the elderly cohort and 4.1% (*n* = 3) in the non‐elderly cohort (*p* = 0.4, 95% CI −5 to 7).

### 3.3 Postoperative outcomes (Table 2)

The median length of stay (LOS) was 7 days in the elderly cohort and 6 days in the non‐elderly cohort (*p* = 0.007, 95% CI −7 to −1). The overall complication rate was 30.3% (*n* = 27) in elderly patients and 26% (*n* = 19) in non‐elderly patients (*p* = 0.2, 95% CI −18 to 9). In the elderly cohort, the majority of complications were surgical site infections (SSI) (16.9%, *n* = 15). The incidence of SSI was 9.6% (*n* = 7) in non‐elderly patients (*p* = 0.09, 95% CI −18 to 3). Major morbidity was defined as Clavien‐Dindo grade III complications or above. In elderly patients, the incidence of major morbidity was 6.7% (*n* = 6) compared with 12.3% (*n* = 9) in non‐elderly patients (*p* = 0.1, 95% CI −3 to 15). SSI was defined as a clinical diagnosis of superficial infection at an incision site that required treatment with antimicrobial therapy. Cardiorespiratory complication was defined as an acute cardiac or respiratory condition that required medical intervention, such as acute coronary syndrome, lower respiratory tract infection or acute pulmonary oedema.

The anastomotic leak (AL) rate was 2.2% (*n* = 2) in the elderly cohort and 6.8% (*n* = 5) in the non‐elderly cohort (*p* = 0.07, 95% CI −2 to 11). All ALs were diagnosed by cross‐sectional imaging which was performed on the basis of clinical suspicion for AL. The two ALs observed in elderly patients occurred following a sigmoid colectomy and right hemicolectomy. In the non‐elderly cohort, the five ALs observed occurred following a high anterior resection for rectosigmoid malignancy and four low anterior resections for low rectal malignancies post‐neoadjuvant chemoradiotherapy. All ALs required surgical intervention except for one AL post‐low anterior resection which was managed by endoscopic clipping of a small anastomotic defect.

The 30‐day abdominal reoperation rate in elderly patients was 5.6% (*n* = 5). Apart from the two previously mentioned ALs, abdominal reoperation occurred in this cohort for postoperative small bowel obstruction (1.1%, *n* = 1), stoma stenosis (1.1%, *n* = 1) and fascial dehiscence (1.1%, *n* = 1). The 30‐day abdominal reoperation rate in non‐elderly patients was 9.6% (*n* = 7) (*P* = 0.2, 95% CI −4 to 12), which occurred for the four previously mentioned ALs, fascial wound dehiscence (2.7%, *n* = 2) and stoma necrosis (1.4%, *n* = 1). One 30‐day mortality occurred in the elderly cohort (*n* = 1.1%) from postoperative acute cardiac failure, while no 30‐day mortalities occurred in non‐elderly patients (*p* = 0.2, 95% CI −4 to 1).

### 3.4 Surgical specimen quality (Table 2)

The median lymph node harvest for oncological resections was 14 nodes in both elderly and non‐elderly patients. No positive margins were recorded in the elderly cohort, while one non‐elderly patient has positive margins at the pre‐sacral fascia following a low anterior resection for what remained a T4N2 tumour at pathological staging following neoadjuvant chemoradiotherapy (*p* = 0.1, 95% CI −1 to 4).

### 3.5 Octogenarian subgroup

A subsequent analysis of perioperative and postoperative outcomes was performed in patients aged 80 years and older at the time of surgery (*n* = 20). The ASA grade was 3 in 50% (*n* = 10) of octogenarians and 70% (*n* = 14) were male. The overall complication rate in this group was 35% (*n* = 7), of which only two complications (10%) met the definition of major morbidity, one of which was an AL post‐right hemicolectomy that required reoperation and the other was stoma stenosis that required surgical revision. The incidence of AL in this cohort was thus 5% (*n* = 1). No patient in this group developed postoperative cardiac or respiratory complication. The incidence of SSI was 15% (*n* = 3). No mortalities were observed in octogenarians. The median LOS was 11 days. When compared with the non‐elderly cohort, no statistically significant differences in overall complication (*p* = 0.2, 95% CI −31 to 13), major morbidity (*p* = 0.39, 95% CI −14 to 18), reoperation (*p* = 0.47, 95% CI −15 to 14) or mortality (*p* = 1.0) was observed.

## 4 DISCUSSION

As the population continues to age, colorectal surgeons will increasingly be required to treat older, frailer patients, highlighting the need for effective treatment strategies in this challenging patient population.^7^ Robotic surgery continues to gain popularity amongst colorectal surgeons, with the inherent ergonomic advantages offered by robotic surgical platforms allowing one to potentially overcome the limitations of laparoscopic surgery as a result of rigid instrumentation and unstable views.^14^ However, in the presence of the ever‐growing elderly population, it is important to ascertain as to whether or not new treatment strategies, such as RCRS, are safe and effective in this cohort. Despite this, there is currently limited data that specifically evaluates outcomes in RCRS in older patients, with many early studies reporting on patient populations that were relatively young with low ASA scores and good performance status.^20^, ^21^, ^22^ Furthermore, some have expressed resistance to the utilisation of robotic surgery in elderly patients due to the risk of prolonged general anaesthesia as a result of a longer operating time and the potential stress caused by hypercapnia due to pneumoperitoneum and Trendelenberg position.^19^ However, it is important to note that such criticisms were also frequently aimed at LRCS initially when compared against open surgery, with subsequent level 1 evidence demonstrating significant reduction in morbidity for elderly patients.^4^

While it has been observed that the reduced systemic insult from minimally invasive surgery may be particularly beneficial in an elderly population that often has lower physiological reserve and therefore may be less able to withstand the physiological stress of open surgery, to date this has largely been demonstrated in the laparoscopic literature, with only a handful of previous studies having specifically examined the impact of older age on outcomes in RCRS.^4^, ^11^, ^20^, ^21^, ^22^ Westrich et al retrospectively evaluated 58 patients over the age of 80 that underwent RCRS for CRC in a single institution over a five‐year period, observing a major morbidity rate of 12% and 90‐day mortality rate of 1.7%, with overall and disease‐free survival of 81% and 87.3% respectively. The authors determined that RCRS is feasible in this demographic, however this study was limited by the absence of a control group.^21^ Su et al. similarly demonstrated favourable findings when retrospectively comparing outcomes for 30 elderly patients with 126 non‐elderly patients that underwent RCRS for rectal cancer, showing a statistically significant reduction in postoperative complications in elderly patients with no mortalities in either group, however this study was limited by a relatively modest cohort of elderly patients.^22^ Oldani et al reported on age‐related outcomes for the first 50 cases of RCRS in a single institution (22 elderly vs. 28 non‐elderly), and similarly observed no statistically significant differences in a wide range of postoperative outcomes, determining that age alone should not be considered an exclusion criterium for RCRS.^20^ It has similarly been demonstrated that age does not negatively impact outcomes in robotic gynaecological and urological surgery.^1^ To our knowledge, the current study is the largest to specifically examine outcomes in RCRS between elderly and non‐elderly patients.^20^, ^21^, ^22^

In the current study, perioperative and postoperative outcomes were compared between an elderly and non‐elderly cohort in a RCRS programme over a five‐year period. The elderly group was more frail and comorbid, with a significantly higher proportion of these patients having an ASA grade of III. Despite this, perioperative and postoperative outcomes compared favourably with non‐elderly patients, with no statistically significant difference in postoperative morbidity or mortality. Only one patient developed a postoperative cardiac complication, with no intraoperative cardiopulmonary events reported. A statistically significant difference in inpatient length of stay was noted, however this only amounted to a median length of stay that was 1 day longer than the non‐elderly cohort. Elderly patients also trended towards having more advanced disease than non‐elderly patients in cases performed for CRC, with the proportion of patients with T4 disease being more than twice that of the younger patient demographic. This may suggest that elderly patients potentially posed a greater operative technical challenge. Despite this, intraoperative and surgical specimen outcomes were comparable with the younger patient cohort, with no statistically significant difference in conversion to open surgery, estimated blood loss, lymph node yield or R0 resections. These results serve to demonstrate that RCRS is safe, feasible and effective in elderly patients, with comparable perioperative and postoperative outcomes when compared with a younger, less comorbid patient cohort. This suggests that patient age should not act as a contraindication to a robotic approach for colorectal disease. These findings are important, as only a small number of studies have specifically investigated the impact of age on outcomes in patients undergoing RCRS.^20^, ^21^, ^22^ It is also interesting to note that more than half of patients that underwent RCRS in our institution were in the elderly demographic, reflecting the current climate of an increasingly older population requiring complex major intra‐abdominal surgery.

It has previously been reported that major elective intra‐abdominal surgery in octogenarian patients poses a particularly high risk of morbidity and mortality, with postoperative mortality as high as 11.4% in this cohort.^24^, ^25^ For this reason, a subgroup analysis was performed focussing on patients aged 80 years and older. Half of these patients were deemed to have had severe systemic disease on anaesthetic preoperative assessment. Despite this, no statistically significant difference was seen in octogenarians when compared with the non‐elderly patient cohort, suggesting that RCRS is a safe approach in this particularly vulnerable patient demographic. A statistically significant difference in median inpatient LOS was noted, being 5 days longer than that of non‐elderly patients. However, given the low rate of morbidity in octogenarians, it is likely that a significant factor in this increase in LOS related to convalescent care requirements upon being deemed medically fit for discharge.

The current study is not without limitations. The study was conducted in a single centre, was retrospective in nature and focussed on a relatively modest cohort of patients which may affect the power of the study to detect difference in outcomes. Only short‐term outcomes were examined, thus conclusions regarding the impact of age on long‐term oncological outcomes and survival cannot be determined. There also exists the potential of selection bias, whereby fitter and less comorbid elderly patients may have been more readily offered surgical intervention than frailer and higher‐risk elderly patients. The risk of type I error and type II error is also a potential limitation of the analysis in this study. Nonetheless, these findings are of importance given that there is a paucity of literature currently available that assesses the safety and feasibility of RCRS in this particularly vulnerable and ever‐expanding patient population. It is important to gain further clarity on this matter as the ability to offer safe and effective complex intra‐abdominal surgery for elderly patients is increasingly required of colorectal surgeons.

In conclusion, the current study demonstrates that RCRS in elderly patients is safe, feasible and effective, with acceptable perioperative and postoperative outcomes that largely do not differ significantly with a younger, less comorbid patient population. Thus, older age should not be considered to be a specific exclusion criterium for undergoing RCRS. To our knowledge, this study represents the largest in the literature to specifically examine outcomes in elderly patients undergoing RCRS.

## CONFLICTS OF INTEREST

The author declares that there is no conflict of interest that could be perceived as prejudicing the impartiality of the research reported.

## INFORMED CONSENT

Informed consent was obtained from all patients for being included in the study.

## CODE AVAILABILITY

Statistical analysis was performed using IBM SPSS version 24 (SPSS Inc, Chicago, IL, USA).

## Data Availability

The data used for this study is available on reasonable written request to the authors.
